# Photoacoustic Imaging for Assessing Tissue Oxygenation Changes in Rat Hepatic Fibrosis

**DOI:** 10.3390/diagnostics10090705

**Published:** 2020-09-17

**Authors:** Mrigendra B. Karmacharya, Laith R. Sultan, Brooke M. Kirkham, Angela K. Brice, Andrew K.W. Wood, Chandra M. Sehgal

**Affiliations:** 1 Department of Radiology, Perelman School of Medicine, University of Pennsylvania, 3620 Hamilton Walk, Philadelphia, PA 19104, USA; Mrigendra.Karmacharya@pennmedicine.upenn.edu (M.B.K.); lsultan@pennmedicine.upenn.edu (L.R.S.); kirkham.brooke@gmail.com (B.M.K.); 2 University Laboratory Animal Resources, University of Pennsylvania, 3800 Spruce Street, Philadelphia, PA 19104, USA; abrice@upenn.edu; 3 Department of Clinical Studies, School of Veterinary Medicine, University of Pennsylvania, 3800 Spruce Street, Philadelphia, PA 19104, USA; akwood@vet.upenn.edu

**Keywords:** oxygen saturation, hemoglobin concentration, echogenicity, angiogenesis, hypoxia

## Abstract

Chronic liver inflammation progressively evokes fibrosis and cirrhosis resulting in compromised liver function, and often leading to cancer. Early diagnosis and staging of fibrosis is crucial because the five-year survival rate of early-stage liver cancer is high. This study investigates the progression of hepatic fibrosis induced in rats following ingestion of diethylnitrosamine (DEN). Changes in oxygen saturation and hemoglobin concentration resulting from chronic inflammation were assayed longitudinally during DEN ingestion by photoacoustic imaging (PAI). Accompanying liver tissue changes were monitored simultaneously by B-mode sonographic imaging. Oxygen saturation and hemoglobin levels in the liver increased over 5 weeks and peaked at 10 weeks before decreasing at 13 weeks of DEN ingestion. The oxygenation changes were accompanied by an increase in hepatic echogenicity and coarseness in the ultrasound image. Histology at 13 weeks confirmed the development of severe fibrosis and cirrhosis. The observed increase in PA signal representing enhanced blood oxygenation is likely an inflammatory physiological response to the dietary DEN insult that increases blood flow by the development of neovasculature to supply oxygen to a fibrotic liver during the progression of hepatic fibrosis. Assessment of oxygenation by PAI may play an important role in the future assessment of hepatic fibrosis.

## 1. Introduction

Chronic liver inflammation leading to liver fibrosis and cirrhosis is a major cause of mortality, accounting for 32,000 deaths in the USA alone and more than one million deaths each year worldwide [1]. Pathologically, liver fibrosis is a consequence of a progressive accumulation of extracellular matrix proteins in the liver parenchyma that results from chronic liver tissue inflammation [2]. A multitude of etiologies such as viral hepatitis (mostly from hepatitis B and C virus), alcoholic and non-alcoholic hepatic injuries, or toxin/drug-induced, metabolic, and autoimmune diseases that trigger reiterative damage to liver parenchyma have been implicated in liver fibrosis [3].

Early diagnosis and management of advanced stages of fibrosis can prevent complications and death, hence prompt diagnosis and staging of fibrosis is crucial [4]. We have shown earlier that B-mode ultrasound can be used for accurate noninvasive fibrosis assessment [5]. Historically, liver biopsy has been used as the diagnostic tool in a variety of chronic liver diseases, and it has remained one of the most frequently performed procedures to evaluate the inflammatory grade and fibrotic stage in chronic liver diseases [6]. However, liver biopsy has limited diagnostic accuracy and reliability, and liver biopsy as a routine diagnostic tool has remained controversial [7]. Several noninvasive diagnostic imaging approaches are also available to detect chronic liver diseases and grade liver fibrosis [8]. These include contrast-enhanced multiphasic computed tomography (CT), positron emission tomography (PET), multiparametric magnetic resonance imaging (MRI), and contrast-enhanced ultrasound (CEUS). In particular, MRI has been used to assess liver oxygenation noninvasively and to detect changes in oxygenation level in liver parenchyma [9]. Despite significant progress, concerns remain: risk associated with cumulative radiation exposure during CT [10], limited spatial and temporal resolution in PET [11], system complexity and susceptibility to artifacts from motion and organ pulsation, and high cost of magnetic resonance imaging [12].

Photoacoustic imaging (PAI) for noninvasive optical contrast sensing with acoustic resolution has gained substantial attention in the past two decades [13]. Its use has progressed significantly as a molecular imaging tool and it offers greater specificity than conventional ultrasound imaging [14]. PAI has been used to predict tumor recurrence and to monitor therapy in glioblastoma [15]. PAI has the ability to detect endogenous light-absorbing chromophores such as hemoglobin and allows visualization of microvasculature and blood oxygenation [16]. PA signals obtained from oxy- and deoxy-hemoglobin can be used for imaging vascular structures, hemoglobin oxygen saturation (sO_2_), blood flow velocity, and the metabolic rate of oxygen [17]. The oxy- and deoxy-hemoglobin absorb light differently at different wavelengths, enabling PAI to generate a high-resolution parametric map of the oxygen saturation of blood noninvasively.

Chronic liver injuries develop a severe, hypoxic microenvironment in hepatic cells, which activate expression of hypoxia-inducible factors. Activation of these factors is critical in the development of liver fibrosis [18]. Expression of hypoxia-inducible factor-1α upregulates the expression of multiple proinflammatory and proangiogenic cytokines, vascular endothelial growth factors, and angiopoietins [19]. The development of a hypoxic cellular environment during hepatic fibrosis leads to inflammation and a demand for greater oxygenation, in turn leading to angiogenesis and increased supply of oxygen through blood flow [20]. Based on these observations, we hypothesize that the increase in blood flow during the progression of liver fibrosis increases tissue oxygenation with an associated enhancement in PA signals. In this study, changes in oxygen saturation and hemoglobin concentration during gradual progression of liver fibrosis were assessed by PAI in a rat model. In addition, the tissue changes associated with hepatic fibrosis were monitored by grayscale B-mode ultrasound imaging to confirm longitudinal changes in the liver [21,22,23]. Histology performed on liver tissue acquired at the end of the study was used to further confirm hepatic fibrosis. We anticipate that assessment of oxygen saturation and hemoglobin concentration by PAI will allow direct assessment of physiological and functional characteristics of the liver in real time and enhance diagnosis of fibrosis based primarily on observing morphological changes using sonography or other forms of imaging.

## 2. Materials and Methods

### 2.1. Animals

The study was approved by the Institutional Animal Care and Use Committee (IACUC number 804998, approved on 26 September 2017). Sixteen adult male Wistar albino rats (age = 6 weeks, 200 to 250 g; Charles River Laboratories, Wilmington, MA, USA) were accommodated in metabolic cages under controlled environmental conditions (25 °C and a 12 h light/dark cycle). All imaging was performed during the light cycle. Animals had free access to standard powdered rat pellet food and tap water unless otherwise indicated. Liver fibrosis was induced in 12 rats by ingestion of 0.01% diethylnitrosamine (DEN solution, Sigma Aldrich, St. Louise, MO, USA) in water ad libitum for 13 weeks. The control group (*n* = 4) received no DEN. At the completion of each experiment, the rats were euthanized by CO_2_ asphyxiation. For euthanasia, the rats were kept in a tightly closed chamber where CO_2_ was introduced from a compressed cylinder for 10 min. The death of the rats was confirmed by ascertaining their cardiac and respiratory arrest. A necropsy was performed immediately after euthanasia.

### 2.2. Photoacoustic Imaging and Analysis

In each animal, PA signals for hemoglobin oxygen saturation (sO_2_) and hemoglobin concentration (Hb) in the right and left lobes of the liver were measured (Vevo LAZR photoacoustics imaging system; FUJIFILM VisualSonics, Toronto, ON, Canada). For imaging, the rats were anesthetized under inhalational isoflurane vaporizer (VetEquip Inc., Livermore, CA, USA) supplied with isoflurane vapor (2 to 4%) and oxygen gas (100 to 200 mL/min). The rats were placed supine on the imaging platform during the whole experiment, while regulated amounts of isoflurane and oxygen were continuously supplied via a nose cone.

Data were acquired at the baseline (before initiating DEN ingestion), and at 5, 10, and 13 weeks later using a 9–18 MHz broadband transducer with axial and lateral resolution of 100 and 235 μm, respectively. The Oxy-Hemo mode was used for PAI, which measures oxygenation saturation and hemoglobin levels in oxygenated and deoxygenated blood in the tissue area or volume. The following imaging presets were used for PAI: PA Gain = 35 dB, Persistence = 8, Wavelength = 750/850 nm. These settings were kept constant for all the groups at different time points. Four to six PA images were acquired in each lobe; they were optimized for each animal at the baseline and the same imaging parameters and time gain control were used in the subsequent longitudinal studies. PA signal in each image was analyzed quantitatively (VisualSonics VevoLAB software, Toronto, ON, Canada) for the following four parameters:

1. sO_2_ average (sO_2_Av)—the average blood oxygen saturation, which is the sum of all oxygenated pixels within the region of interest (ROI), divided by the oxygenated and deoxygenated pixels. Thus sO_2_Av measures the average percentage of oxygen saturation of the pixels with PA signal within the ROI.
2. sO_2_ average total (sO_2_AvT)—the average value calculated from all pixels within the ROI, representing the percent oxygenation of the total tissue within the ROI.
3. HbT—the average hemoglobin concentration calculated from the pixels with hemoglobin signal within the ROI.
4. HbT average (HbTAv)—the total hemoglobin concentration calculated from all pixels within the ROI.

The quantified values (mean ± SEM) for each parameter were averaged and normalized with the baseline values and analyzed.

### 2.3. Sonographic Imaging and Analysis

At each of the four time points (baseline, 5, 10, and 13 weeks), 4 to 6 B-mode sonographic images (13–24 MHz transducer MS250; FUJIFILM VisualSonics, Toronto, ON, Canada) were obtained of the right and left lobes of each rat’s liver in transverse and sagittal anatomic planes immediately after PAI. Imaging presets (gain = 18 dB; high sensitivity; 100% power; transmit frequency 21 MHz; high line density) and time gain compensation were optimized and standardized. Ultrasound images were acquired by the same experienced operator at all time points. Image texture features were analyzed using MaZda software (Technical University of Lodz, Lodz, Poland) [24]. The measurements were made on 4 to 6 ROIs in each image and included the following texture features [25]:

- echogenicity (brightness level)—the first-order global distribution of intensity values in the grayscale images
- heterogeneity (variance)—a measure of how far from the mean the gray-level values in the image are distributed
- contrast—how much difference, or definition, there is between gray-level values of different objects in the image
- entropy—measures the randomness or inhomogeneity of the pixel distribution with respect to length or orientation, with a higher value for a more random distribution; it measures disorderliness in the image
- run length—a matrix that records runs of adjacent pixels having the same gray-level value, over multiple directions
- absolute gradient—measures the gradient-based features from each pixel compared with the neighborhood pixels. The absolute gradient of an image computes the spatial variation of gray-level values across the image

The computed values (mean ± SEM) for each parameter were averaged over all ROIs. The values were then normalized with respect to the baselines and analyzed. Measurements were also made on the ROIs drawn on the liver–right kidney interfaces to compare changes in the echogenicity of the liver with respect to those of the kidney. An example of ROIs used for assessing liver texture and hepatorenal index is shown in Supplementary Figure S1. Quantitatively, the hepatorenal index (HRI) was calculated and analyzed.

### 2.4. Histochemical Staining and Analysis

Following imaging and necropsy at the 13-week time point, samples were taken from the right and left liver lobes for histologic examination. They were preserved in 10% phosphate-buffered formalin for 48 to 72 h, transferred to 50% ethanol for 24 h, embedded in paraffin, and finally processed for histological examination with hematoxylin and eosin (H&E) and trichrome staining. The slides were examined under a microscope (Olympus BX51, Olympus America Inc., Melville, NY, USA) and the presence of fibrosis, inflammation, and necrosis was recorded. Collagen is observed as pale pink on H&E stained tissue, and the fibrous tissue was seen as blue-stained fibers on trichrome stain. Each histologic section was graded according to the METAVIR scoring system for hepatic fibrosis: F0, no fibrosis; F1, portal fibrosis without septa (mild); F2, portal fibrosis with rare septa (moderate); F3, numerous septa without cirrhosis (severe); and F4, cirrhosis [26]. Collagen deposits were calculated by Masson’s trichrome method using ImageJ software as described previously [27].

### 2.5. Statistical Analysis

Boxplots displaying the five-number summary of a set of data (the minimum, first quartile, median, third quartile, and maximum) were calculated and plotted. Statistical analyses of the PA and sonographic data, given as a mean ± SEM for each ROI, were performed using one-way analysis of variance (ANOVA), assuming the null hypothesis that there is no increase in signal between the groups and equality between the means. The ANOVA was followed by a post hoc Tukey’s test. Statistical significance was represented as *** for *p* ≤ 0.001, ** for *p* ≤ 0.01 and * for *p* ≤ 0.05, and ns (not significant). Prior to the ANOVA test, the Shapiro–Wilk test was performed to test data normality. *p*-value > 0.05 was used to accept the data as normally distributed.

## 3. Results

### 3.1. Photoacoustic Imaging

**Figure 1:**
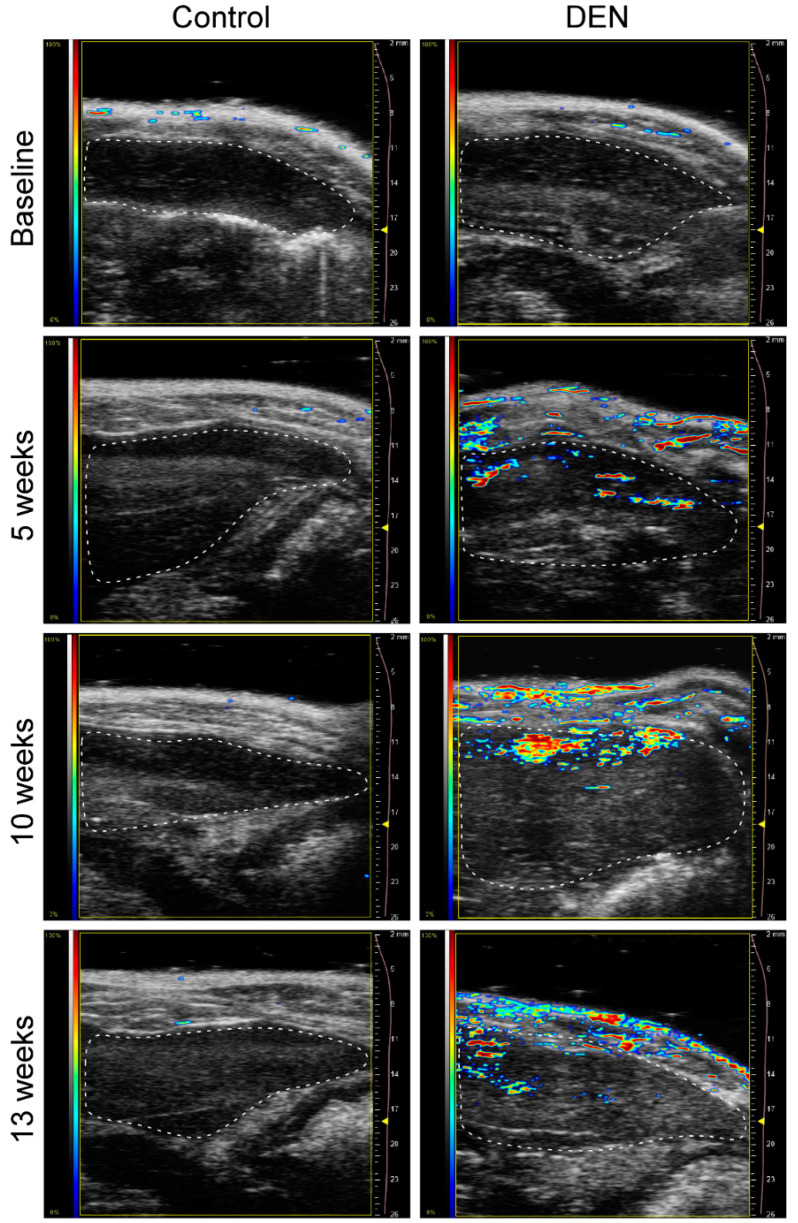
PA (photoacoustic) images of the livers of the control versus DEN (diethylnitrosamine)-ingested rats. The PA signals obtained are superimposed on the sagittal ultrasound images of the left liver lobe, the borders of which have been outlined. PA signal acquired from the OxyHemo mode measures PA signal at two wavelengths, 750 nm and 850 nm. PAI (photoacoustic imaging) quantifies and maps hemoglobin concentrations from the optical absorption coefficients and molar extinction coefficients of the oxygenated and deoxygenated hemoglobin at the given wavelength. The total hemoglobin concentration and oxygen saturation are derived from oxygenated and deoxygenated hemoglobin concentration. The displayed images were acquired in the OxyZated mode of the Vevo LAZR platform and represent oxygen saturation. At baseline, the image was made prior to the ingestion of DEN; images were obtained 5, 10, and 13 weeks respectively after DEN administration. The deep red color in the color bar represents the highest (100%) and deep blue color the lowest (0%) PA signal. Note the qualitative increases in PA signals following DEN therapy and onset of hepatic fibrosis. Control rats showed no increase in PA signals over time.

Visual qualitative assessments of the liver exhibited only a minimal PA signal at the baseline, but it increased after 5 and 10 weeks’ administration of DEN (Figure 1). Figure 1 displays the “OxyZated” image of the Vevo LAZR platform, which represents oxygen saturation. Images can be acquired in the “HemoMeaZure” mode for hemoglobin concentration. An example of images acquired in the “OxyZated” and “HemoMeaZure” mode is shown in Supplementary Figure S2. There was a noticeable increase in the PA signals (both oxygen saturation and hemoglobin concentration) in liver tissue at 5 weeks which became more pronounced by week 10. At the 13th week, however, there was a decrease in PA signals compared with the 10th week. PA signal in the control rats did not change over time and remained similar to that observed at the baseline. Notably, contrary to the control rats, DEN-ingested rats showed augmented PA signal in skin in some cases, which increased over time after DEN ingestion from baseline to 13 weeks. It should be noted here that the increased subcutaneous PA signal after 5, 10, or 13 weeks of DEN ingestion was not uniformly observed in all the cases. While some cases showed increased PA signal from skin (Figure 1) others showed either no signal or much less signal in skin for 5 10, or 13 week DEN-ingested groups (Supplementary Figure S3). The increased PA signal after 5, 10, or 13 weeks of DEN administration is likely due to dilated blood vessels and increased blood flow in cutaneous regions caused by DEN-induced liver disease. It has previously been reported that dilation of blood vessels near the skin surface is one of the cutaneous manifestations of liver disease, including viral hepatitis and cirrhosis [28].

**Figure 2:**
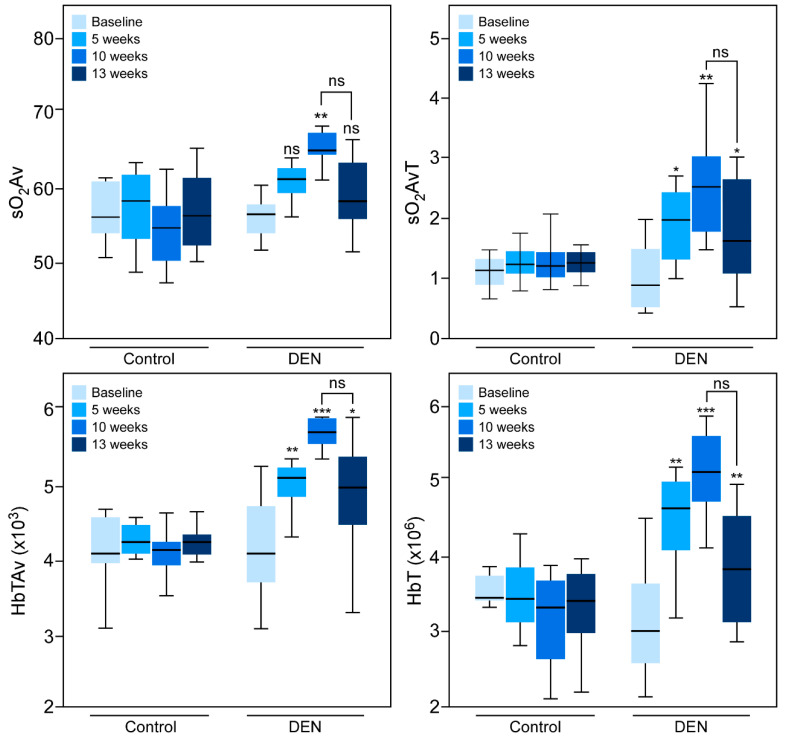
Quantitative analyses of hepatic PA signals. Values of oxygen saturation (average = sO_2_Av and average total = sO_2_AvT) and hemoglobin concentration (average = HbTAv and average total = HbT) were obtained for 12 rats, prior to the start of DEN consumption (baseline) and 5, 10, and 13 weeks after DEN administration, and then averaged. The mean values (± SEM) were plotted. Note that as the hepatic fibrosis developed there were statistically significant increases in each parameter at the 10-week time point. The ANOVA *p-*values between baseline and 10 weeks for sO_2_Av, sO_2_AvT, HbTAv, and HbT were 0.01, 0.02, 0.0003, and 0.0001, respectively. Control rats showed no increase in these parameters over time. Statistical significance was assigned as * for *p* ≤ 0.05, ** for *p* ≤ 0.01, and *** for *p* ≤ 0.001, and ns for non-significant.

Quantitatively, data in all four groups, namely, baseline, 5, 10, and 13 weeks, for all the parameters (oxygen saturation (sO_2_Av and sO_2_AvT) and hemoglobin concentration (HbTAv and HbT)) were normally distributed. The Shapiro–Wilk *p*-values for all the groups for all four PA parameters were > 0.05. Oxygen saturation (sO_2_Av and sO_2_AvT) and hemoglobin concentration (HbTAv and HbT) values increased significantly following 10 weeks of DEN treatment (Table 1 and Figure 2).

Further analyses showed that sO_2_Av increased by 12.81% (± 0.03), sO_2_AvT by 92.04% (± 0.29), HbTAv by 29.7% (± 0.06), and HbT by 55.24% (± 0.10) at 10 weeks. The increases in sO_2_AvT_,_ HbTAv and HbT were significant at 5 weeks; sO_2_AvT increased by 53.83% (± 0.25), HbTAv by 22.12% (± 0.05), and HbT by 35.31% (± 0.07). At the 13th week, however, the values of all of these parameters were lower than those at the 10th week, although the decrease did not reach statistical significance. Notably, the values of sO_2_Av_,_ sO_2_AvT, and HbTAv at the 13th week were comparable to the baseline; only HbT was significantly higher than the baseline. The value of HbT increased by 17.78% (± 0.07) compared with the baseline at 13 weeks. Individual data points of each animal are shown in Supplementary Figure S4. None of these parameters changed over time in control rats. The differences in sO_2_Av, sO_2_AvT, HbTAv and HbT from baseline to 13 weeks were not statistically significant.

### 3.2. Sonographic Imaging of Liver Tissue and Assessment of Texture Features

**Figure 3:**
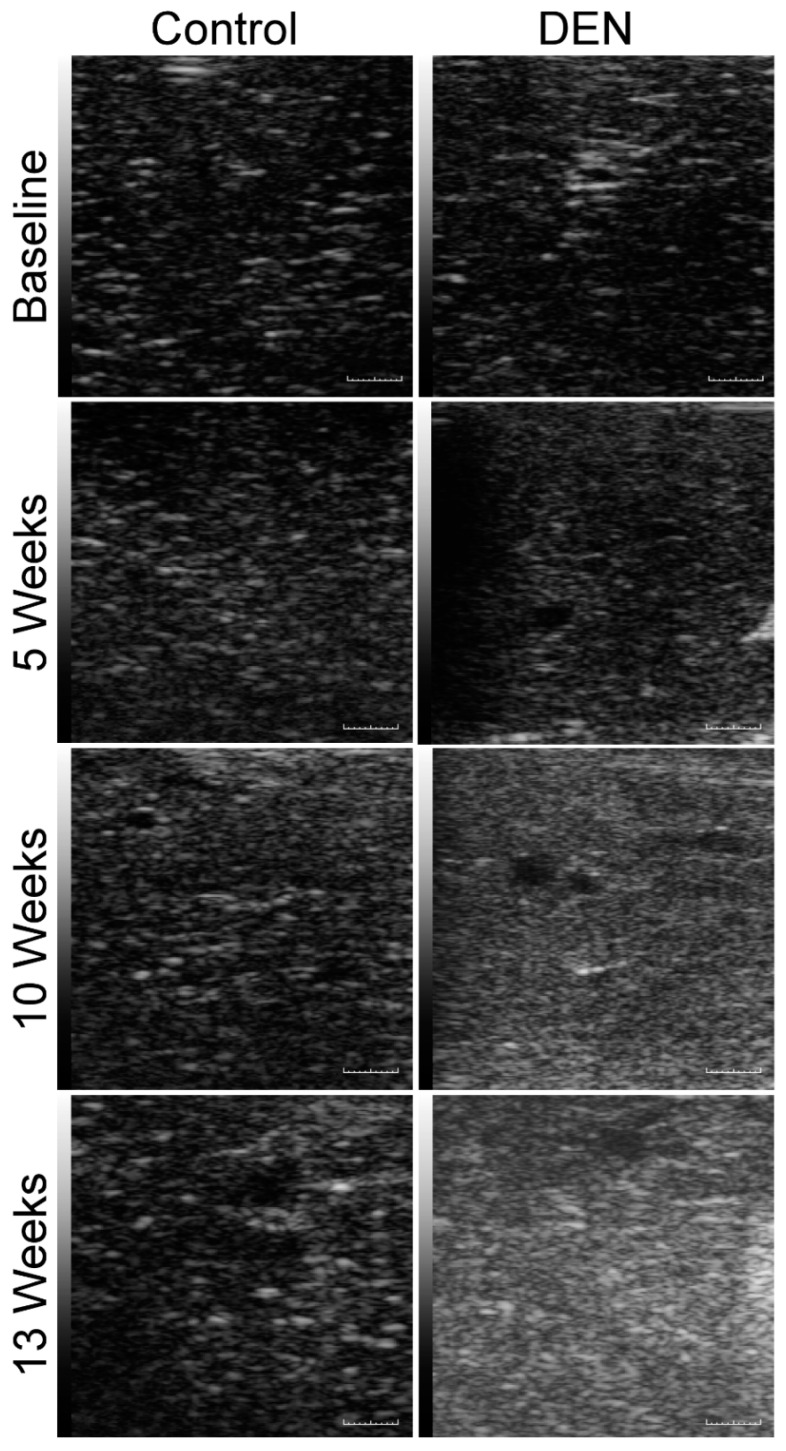
Qualitative assessment of echogenicity of sagittal B-mode ultrasound images. Sagittal B-mode sonograms of the middle lobe of rat livers were obtained either prior to the ingestion of DEN (baseline) or 5, 10, and 13 weeks after its administration. Note that as the hepatic fibrosis developed there were statistically significant increases in echogenicity of the liver tissue 10 and 13 weeks after the commencement of DEN administration compared to the control rats. Control rats showed no increase in echogenicity over time and remained comparable to the baseline. Scale bar = 1 mm.

**Figure 4:**
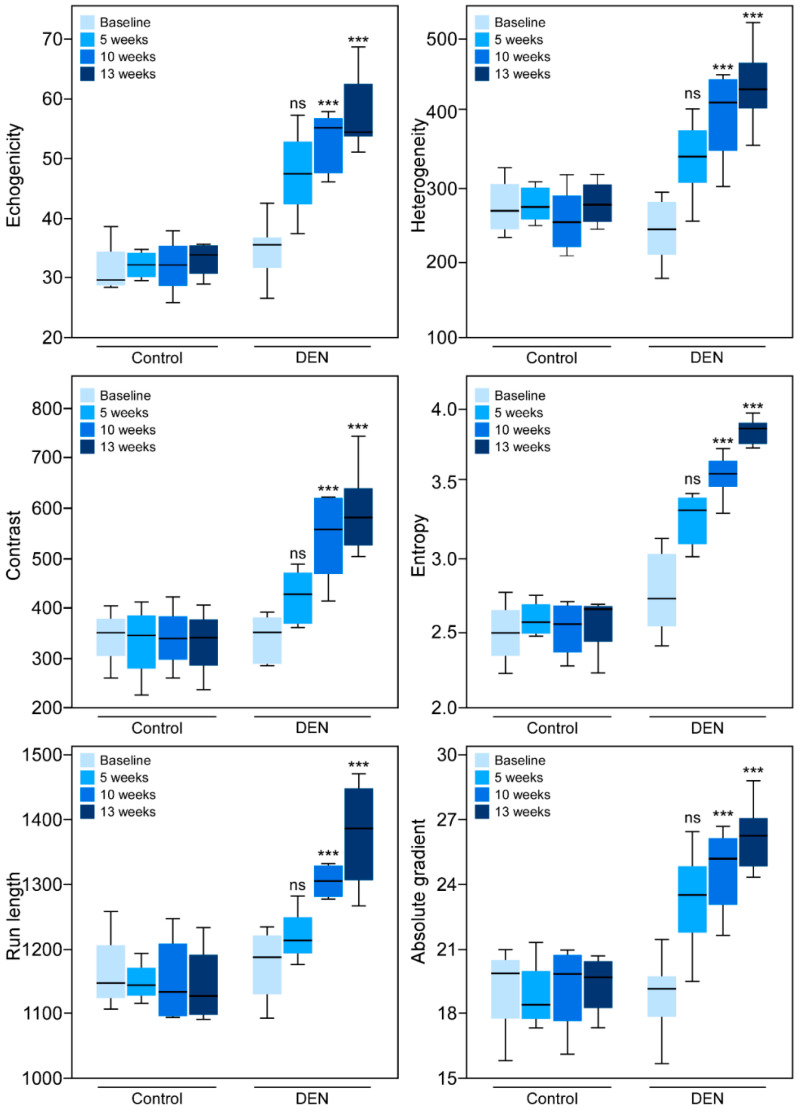
Analyses of texture features in hepatic B-mode ultrasound images. Boxplots showing distribution of six texture feature values (echogenicity, heterogeneity, contrast, entropy, run length, and absolute gradient) obtained from ultrasound image analyses taken either prior to the ingestion of DEN (baseline) or 5, 10, and 13 weeks after DEN administration. The texture features were determined from the images exported from the scanner in tiff format. Distributions of the data points with the minimum, first quartile, median, third quartile, and maximum are shown for each group. The horizontal line inside the box is the median. Note that as the hepatic fibrosis developed there were statistically significant increases in each of the parameters 10 and 13 weeks after the commencement of DEN administration. Control rats showed no increase in the texture features over time. The ANOVA *p*-values between baseline and 10 weeks and baseline and 13 weeks for all the six texture features were less than 0.0001. Statistical significance was assigned as *** for *p* ≤ 0.001, and ns for non-significant.

Qualitative visual inspection of the sonographic images from the baseline up to 13 weeks after administration of DEN reveals that the liver tissue became hyperechoic over time (Figure 3). In the DEN-treated animals, the echogenicity of the liver tissue increased continually over 13 weeks. Quantitatively, various texture features extracted from the grayscale sonograms of the liver tissue showed a gradual increase over time which was statistically significant from the baseline to 10 and 13 weeks (Table 2 and Figure 4).

Over time, control rats that did not receive DEN did not show any qualitative or quantitative changes in echogenicity or texture features. Prior to comparison of the average values of the texture features between the baseline, 5, 10, and 13 week groups, the values of the texture features echogenicity, contrast, run length, heterogeneity, entropy, and absolute gradient were tested by the Shapiro–Wilk test for normal distribution of the data set. The *p*-values for the Shapiro–Wilk test for all the groups for all texture features were > 0.05.

### 3.3. Sonographic Imaging of Hepatorenal Interface and Assessment of HRI

**Figure 5:**
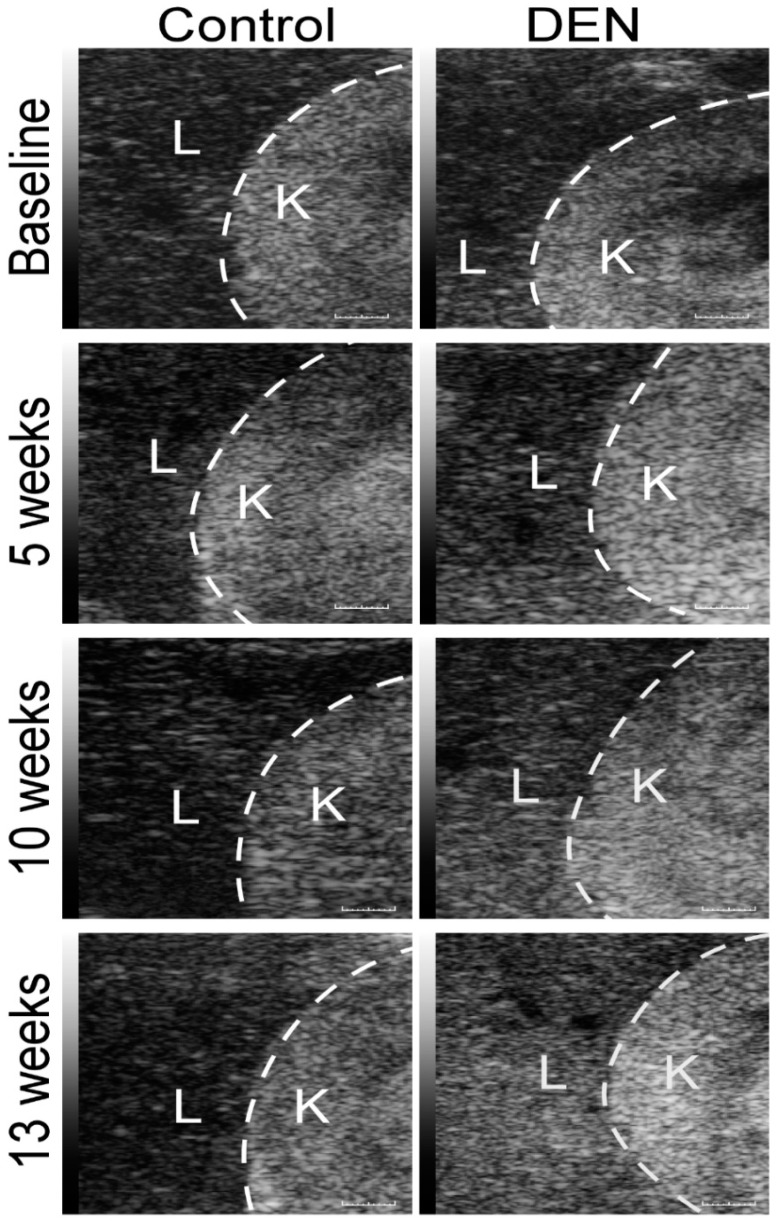
Sagittal B-mode ultrasound images of the right liver lobes of the control versus DEN-ingested rats. The images were made prior to the ingestion of DEN (baseline) and 5, 10, and 13 weeks after its administration. Note the progressive, qualitative increase in the echogenicity of the liver (L) with the development of hepatic fibrosis, so that by 13 weeks it is approaching that of the contiguous right kidney (K). Control rats showed no increase in liver echogenicity, which remained much less than that of the kidney over time. Scale bar = 1 mm.

**Figure 6:**
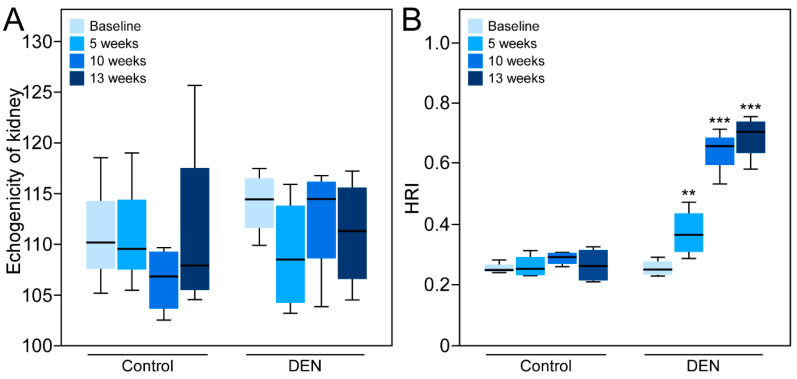
Quantitative analyses of the hepatorenal index (HRI). (**A**) Boxplots showing distribution of echogenicity values of the right kidney obtained from 12 rats prior to the start of DEN consumption (baseline) and 5, 10, and 13 weeks after DEN administration. Echogenicity of kidney of both the control and DEN-fed rats did not change with time. (**B**) Boxplots showing distribution of HRI measurements obtained in 12 rats prior to the start of DEN consumption (baseline) and 5, 10, and 13 weeks after DEN administration. Distribution of the data points with the minimum, first quartile, median, third quartile, and maximum are shown. The horizontal line inside the box is the median. As the hepatic fibrosis developed there were statistically significant increases in HRI. The ANOVA *p*-value for mean values of HRI for baseline versus 5, 10, and 13 week groups were all statistically significant. Control rats showed no increase in HRI over time. Statistical significance was assigned as ** for *p* ≤ 0.01, and *** for *p* ≤ 0.001.

A qualitative visual inspection of the sonographic images at the baseline revealed that the liver tissue was hypoechoic to the kidney (Figure 5). Notably, the echogenicity of kidneys of the control as well as of the DEN-fed rats did not change with time (Figure 6A). In the DEN-treated animals, the echogenicity of the liver tissue increased gradually reaching a maximum at 13 weeks, comparable to that of the kidney. Quantitatively, the echogenicity of the liver tissue also was comparable to the kidney, as represented by the HRI, and showed a statistically significant increase from the baseline to 5, 10, and 13 weeks (Figure 6B). Control rats did not show any increase in HRI and their livers remained hypoechoic to the kidney over time. Before comparing average HRI values between the baseline, 5, 10, and 13 week groups, the dataset was tested for normal distribution using the Shapiro–Wilk test. The Shapiro–Wilk *p*-values for the control and DEN groups from baseline to 13 weeks were all > 0.05.

### 3.4. Histochemical Findings

**Figure 7:**
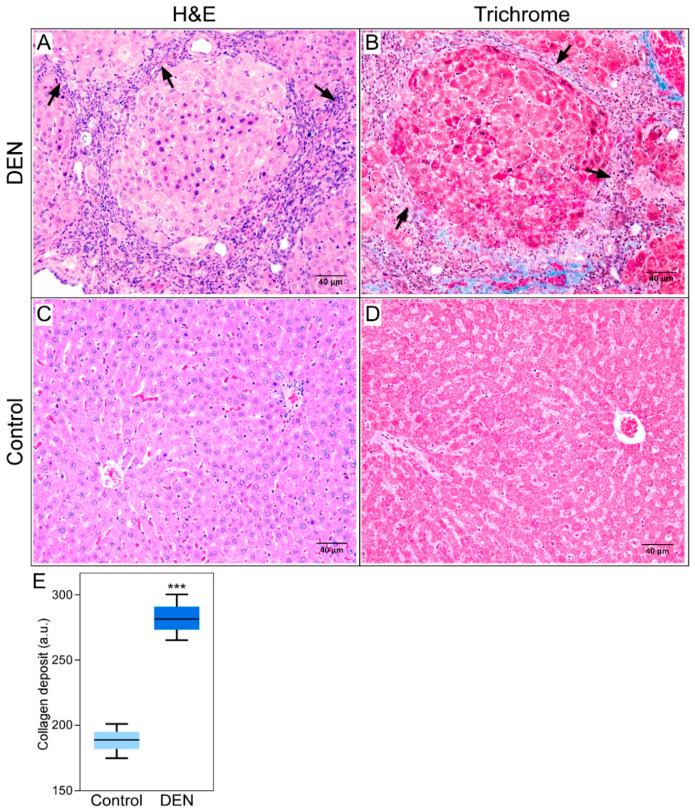
Hepatic histopathology following 13 weeks of DEN treatment. The tissue samples taken from animal subjects 13 weeks after they had started drinking DEN-infused water were processed for histological examination with (**A**) H&E or (**B**) trichrome staining, and imaged under microscope. The H&E and trichrome stains show numerous septa (arrows) formed by deposits of collagen fibers. Liver tissue samples taken from control rats showed normal liver cells without any deposits of collagen fibers in H&E (**C**) or trichrome staining (**D**). The fibrous tissues stained blue on trichrome stain are the deposits of collagen fibers which characterize hepatic fibrosis. Scale bar = 40 μm. Histologic sections (**A**,**B**) were graded METAVIR score of F4, and (**C**,**D**) F0. (**E**) Boxplots showing quantification of collagen deposits in the control and DEN-fed rats. The difference between the mean collagen deposits is statistically significant (*** for *p* < 0.001).

Examination of the liver tissue at 13 weeks showed development of hepatic fibrosis in each of the DEN-treated rats. The livers of the control animals were normal and did not show change over the 13-week period. Each of the DEN-treated animals had a METAVIR score of F3 or F4 (Figure 7A,B), indicating a highly fibrotic/cirrhotic liver, versus F0 for the control animals (Figure 7C,D). The DEN-treated group demonstrated numerous fibrous septa made of deposits of collagen fibers which were not observed in the control animals (Figure 7).

## 4. Discussion

As liver fibrosis advances, the liver parenchyma becomes increasingly hypoxic, inducing an increase in arterial blood supply. In addition, ongoing inflammatory and immune responses during fibrosis are associated with dramatic shifts in tissue metabolism, which are accompanied by a local depletion of nutrients and increased oxygen consumption. It has been reported that the sites of ongoing inflammation and high immune activity can become rapidly depleted of both nutrients and oxygen, which can increase their oxygen demand as much as 50-fold [29]. As an inflammatory response to hypoxia, new blood vessels are formed in the liver parenchyma to help respond to the growing oxygen deficit. Likewise, it has been shown that as an adaptive molecular response to hypoxia, blood cells increase their HbO_2_-carrying capacity and enhance capillary blood supply promoted by improved vascularization along with upregulation of key genes required for these responses, which are largely transcriptionally regulated by HIF-mediated gene expression [30]. These observations suggest a close association between liver fibrosis, inflammatory response, neovascularization and increased oxygen saturation. Furthermore, this implies that progression of liver fibrosis could be assessed by monitoring neo-microvasculature and the hemoglobin oxygen saturation in the liver parenchyma.

DEN is one of the well-established chemical carcinogens that spontaneously induces liver fibrosis in experimental rodent models. It has been shown that a prolonged exposure of rats to DEN gives rise to multistage hepatocarcinogenesis, following the known progression of persistent DNA damage in liver leading to chronic hepatic inflammation, fibrosis, and cancer [31]. Furthermore, it has been reported previously that long-term DEN administration resulted in a 100% success rate in tumor formation [32]. Mechanistically, chronic administration of DEN causes acute liver injury and DNA damage in the hepatocytes, which incites an inflammatory response and secretion of several cytokines and growth factors that promote the proliferation of quiescent hepatocytes carrying DEN-induced mutations [33]. In addition, administration of DEN has been shown to significantly elevate the expression of *hif*-*1α* transcription factor, along with *vegf* and interleukin (*IL*)-*10*, all of which are responsible for angiogenesis and inflammation [34]. These studies demonstrated that DEN-induced hepatotoxicity exhibits close association with hypoxia, inflammation, and angiogenesis in hepatic tissue, all crucial factors in liver fibrosis.

Based on these previous observations, we hypothesized that with the advancement of liver fibrosis, the cellular hypoxic microenvironment induced by DEN ingestion potentiates inflammatory response and necessitates the formation of neovasculature to enhance blood flow in order to supply more oxygen to the severely hypoxic liver cells. Induction of liver fibrosis and cirrhosis following DEN ingestion was confirmed by histology, where formations of numerous fibrous septa, the hallmark of liver fibrosis [35], were clearly visible in the DEN-ingested rats, compared to the normal liver cells in the control rats.

Furthermore, results obtained from PAI showed that with the progression of liver fibrosis, tissue oxygen saturation and hemoglobin concentration increased significantly and peaked at 10 weeks. Not only the global averages of total oxygen saturation and total hemoglobin concentration in the liver tissue were increased, but average oxygen saturation and hemoglobin concentration in the blood vessels were also increased. These observations imply that not only more liver tissue was affected over time with the progression of fibrosis, but also blood carried more oxygen to the affected liver tissue as an inflammatory response. This observation demonstrates that functional changes underlying liver fibrosis could be monitored by assaying changes in hepatic oxygen saturation and hemoglobin concentration. Note that some rats exhibited subcutaneous PA signal after DEN ingestion. Strictly, the PA signal observed in the liver tissue should be compensated for by the overlying skin. Current state-of-the-art imaging does not provide a reliable means to account for differences in light fluence due to skin thickness. These differences, however, are likely to be small due to small differences in the thickness.

Consistent with this study, the increase in liver fibrosis has previously been demonstrated to be accompanied by an increase in hepatic blood flow by Doppler ultrasound imaging [36]. The progression of hepatic microvascular dysfunction in chronically ethanol-treated rats was monitored by measuring an increase in hepatic oxygenation using functional MRI [37]. These observations also support our findings that the progression of liver fibrosis can be assessed by monitoring hepatic tissue oxygenation and hemoglobin concentration by PAI. Photoacoustically derived hemoglobin and oxygen saturation measurements have previously been shown to be good indicators of tumor vascularity [38]. Particularly, sO_2_Av and sO_2_AvT values have been shown to correlate with the expression of inflammatory biomarker cyclooxygenase-2. However, the study did not show any correlation between HbT or sO_2_ signals and expression of cluster of differentiation 31 (CD31) and vascular endothelial growth factor (VEGF).

Interestingly, we observed that all parameters, sO_2_Av, sO_2_AvT, HbTAv, and HbT, peaked at 10 weeks following DEN ingestion but then declined by 13 weeks; however, the decrease did not reach statistical significance. As has been previously reported and also demonstrated in this study (Supplementary Figure S5), DEN induces the formation of aggressive HCC tumors in mice and rats in 12 to 40 weeks [32]. The decrease in PA signals at 13 weeks could reflect the onset of HCC formation, when a greater demand for blood flow from the rapidly growing tumors might outweigh the oxygen and nutrient supply, leading to a reduction in overall tissue oxygenation.

The progressive development of liver fibrosis following DEN ingestion coincided with significant increase in each of the six texture features measured in the sonographic images. Such increases in both hepatic echogenicity and coarseness confirm earlier findings that ultrasound features of hepatic nodularity are reliable predictors of severe fibrosis [39,40]. Interestingly, in contrast to the decline in PA signals between 10 and 13 weeks following ingestion of DEN, the values of all sonographic texture features continued to increase. As hepatic fibrosis developed, these later increases in echogenicity and coarseness could be related to an excessive accumulation of extracellular matrix components in the liver parenchyma [41]. Although increase in liver echogenicity is associated with steatosis as well as fibrosis [42], histological assessment of rat liver tissues in our previous study showed that DEN administration in rodents did not cause liver steatosis [5]. Hence, the accompanying changes in the liver texture features along with increased echogenicity of liver tissue induced by DEN ingestion are more likely due to fibrotic changes rather than steatosis. It is important to note that animals were studied until 13 weeks to document the development of tumors. The 10-week time point was found to be a critical time point for assessing histological changes, which needs to be investigated in more detail in future studies.

With these data, we think that for staging hepatic fibrosis, a combination of PAI with sonographic texture features gives more robust results. Namely, values of both PA and sonographic texture features only slightly higher than those in the baseline indicate the initial fibrotic stage; a much higher PA signal and higher values of texture features indicate fully fibrotic stage; and much higher values in the sonographic texture features with comparatively lower PA signal indicates an advanced cirrhotic stage of fibrosis, where some tumors might have started to form.

## 5. Conclusions

This study demonstrated the ability of PAI to directly assess functional physiological changes in liver tissue during progression of liver fibrosis, establishing that increased oxygen saturation and hemoglobin concentration induced by inflammatory response and formation of hepatic neovasculature during liver fibrosis can be assessed and monitored noninvasively by PA imaging in real time. The use of PAI in combination with sonographic texture feature analysis allows direct assessment of physiological and functional characteristics of the liver, and its use in the future could improve clinical diagnosis of liver fibrosis.

## Figures and Tables

**Table 1:** Values of oxygen saturation (average = sO_2_Av and average total = sO_2_AvT) and hemoglobin concentration (average = HbTAv and average total = HbT) obtained prior to the start of DEN consumption (baseline) and 5, 10, and 13 weeks after DEN administration. Control rats did not receive DEN.

	Groups	Baseline	5 Weeks	10 Weeks	13 Weeks
Control	sO_2_Av	54.7 (± 0.9)	55.5 (± 2.7)	53.3 (± 1.3)	54.3 (± 2.4)
	sO_2_AvT	1.1 (± 0.1)	1.2 (± 0.1)	1.2 (± 0.1)	1.2 (± 0.1)
	HbTAv (×10^3^)	4.1 (± 0.1)	4.2 (± 0.1)	4.1 (± 0.1)	4.1 (± 0.1)
	HbT (×10^6^)	315.1 (± 18.6)	314.3 (± 21.4)	311.2 (± 16.8)	311.4 (± 18.2)
DEN	sO_2_Av	56.1 (± 0.8)	60.6 (± 0.7)	65.2 (± 0.7) **	59.2 (± 1.3)
	sO_2_AvT	1.1 (± 0.2)	1.9 (± 0.2) *	2.7 (± 0.3) **	1.8 (± 0.3) *
	HbTAv (×10^3^)	4.2 (± 0.2)	5.1 (± 0.2) **	5.8 (± 0.3) ***	4.9 (± 0.3) *
	HbT (×10^6^)	313.8 (± 20.4)	445.9 (± 19.8) **	516.3 (± 16.7) ***	384.7 (± 22.5) **

* means *p* ≤ 0.05, ** means *p* ≤ 0.01, and *** means *p* ≤ 0.001.

**Table 2:** Values of various texture features obtained prior to the start of DEN consumption (baseline) and 5, 10, and 13 weeks after DEN administration. Control rats did not receive DEN.

	Features	Baseline	5 Weeks	10 Weeks	13 Weeks
Control	Echogenicity	31.6 (± 2.4)	32.2 (± 1.2)	31.9 (± 2.5)	33.1 (± 1.5)
	Heterogeneity (×100)	2.5 (± 0.2)	2.5 (± 0.1)	2.3 (± 0.3)	2.6 (± 0.1)
	Contrast (×100)	3.3 (± 0.3)	3.2 (± 0.4)	3.3 (± 0.3)	3.2 (± 0.4)
	Entropy	3.01 (± 0.02)	3.02 (± 0.01)	3.01 (± 0.02)	3.01 (± 0.02)
	Run length (×1000)	1.2 (± 0.03)	1.1 (± 0.02)	1.2 (± 0.03)	1.1 (± 0.03)
	Absolute gradient	19.1 (± 0.5)	21.6 (± 0.7)	24.6 (± 0.8)	25.9 (± 0.7)
DEN	Echogenicity	34.7 (± 1.2)	42.5 (± 2.2)	53.1 (± 1.8) ***	57.1 (± 2.8) ***
	Heterogeneity (×100)	2.5 (± 0.2)	3.1 (± 0.2)	3.9 (± 0.3) ***	4.3 (± 0.3) ***
	Contrast (×100)	3.4 (± 0.2)	4.3 (± 0.2)	5.4 (± 0.4) ***	5.9 (± 0.3) ***
	Entropy	3.07 (± 0.02)	3.14 (± 0.02)	3.19 (± 0.01) ***	3.23 (± 0.02) ***
	Run length (×1000)	1.1 (± 0.05)	1.2 (± 0.02)	1.3 (± 0.02) ***	1.4 (± 0.04) ***
	Absolute gradient	19.1 (± 0.5)	21.6 (± 0.7)	24.6 (± 0.8) ***	25.9 (± 0.7) ***

*** means *p* ≤ 0.001.

## Supplementary Materials

The following are available online at https://www.mdpi.com/2075-4418/10/9/705/s1, Supplementary Figure S1. Images showing examples of ROIs drawn for assessing texture features in the liver (left) or at the liver/kidney interface (right); Supplementary Figure S2. An example of PA signals displayed by the same liver tissue in the “OxyZated” and “HemoMeaZure” modes in the Vevo LAZR platform; Supplementary Figure S3. PA images of the livers of the DEN-ingested rats; Supplementary Figure S4. Graphs showing hepatic PA signals of individual rats; Supplementary Figure S5. H&E (left) and Trichrome (right) staining images showing an HCC tumor in rat liver taken after 13 weeks of DEN ingestion.
