# Supplementary material for: Photoacoustic Imaging for Assessing Tissue Oxygenation Changes in Rat Hepatic Fibrosis
Source: Diagnostics (Basel). 2020 Sep 17;10(9):705. doi: 10.3390/diagnostics10090705 (PMC7555416; doi:10.3390/diagnostics10090705)
Supplement: Supplementary file 1 [file diagnostics-10-00705-s001.pdf]

diagnostics

Supplementary Figures

Supplementary Figure S1

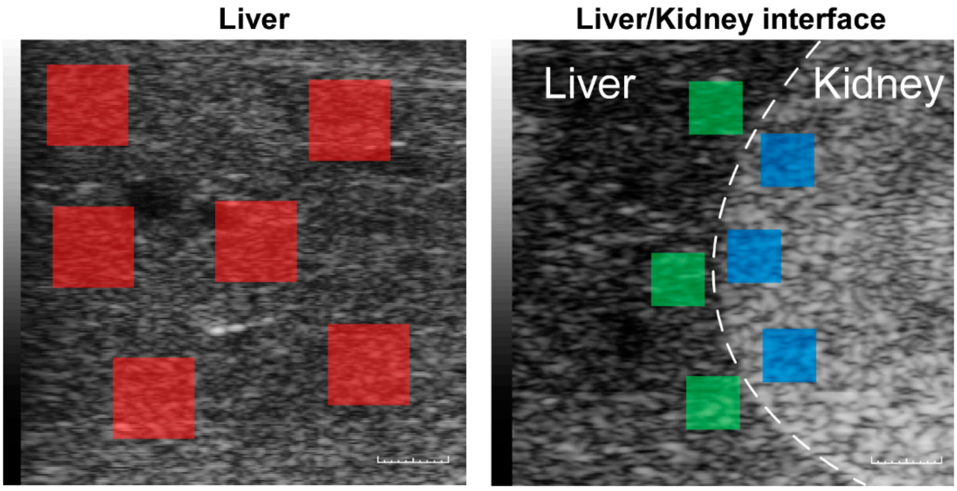

Supplementary Figure S1. Images showing examples of ROIs drawn for assessing texture features in the liver (left) or at the liver/kidney interface (right). Five or six ROIs (represented here by transparent red, green, and blue squares) were drawn in the tissue, and texture features were acquired and analyzed by using MaZda software (Technical University of Lodz, Lodz, Poland). Scale bar = 1 mm.

Supplementary Figure S2

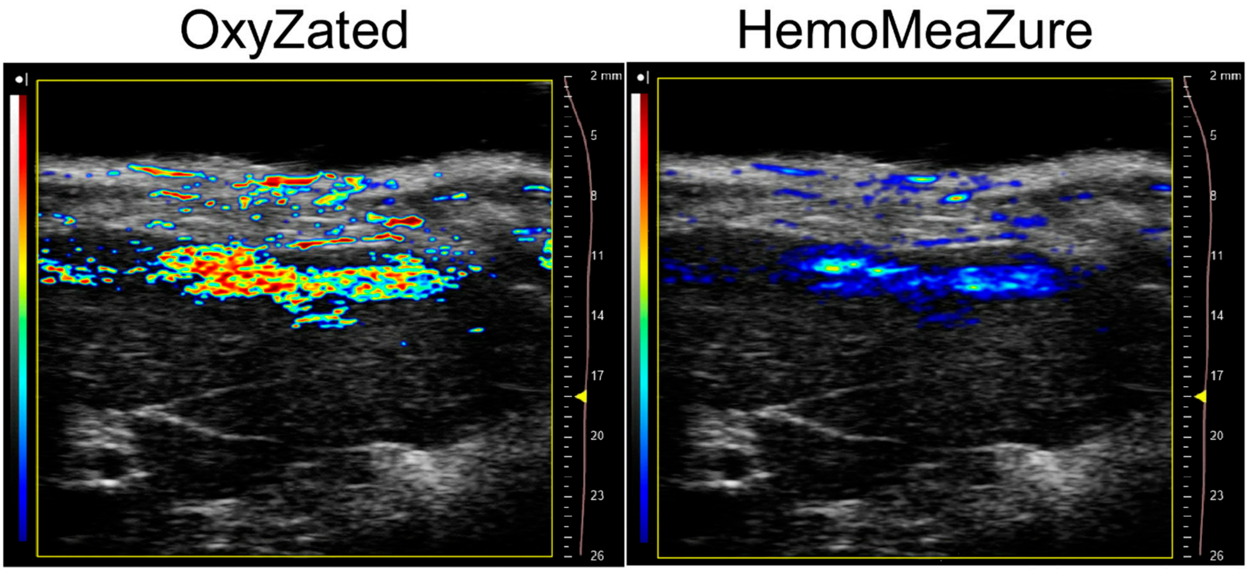

Supplementary Figure S2. An example of PA signals displayed by the same liver tissue in the "OxyZated" and "HemoMeaZure" modes in the Vevo LAZR platform. The images were acquired from a left lobe of a rat liver after 10 weeks of DEN ingestion using the same settings for imaging. The "OxyZated" mode shows "oxygen saturation" and the "HemoMeaZure" mode shows "hemoglobin content" of the liver tissue. Note that the two images are correlated but the image acquired in the "HemoMeaZure" mode did not display as high a sensitivity as that in the "OxyZated" mode.

Supplementary Figure S3

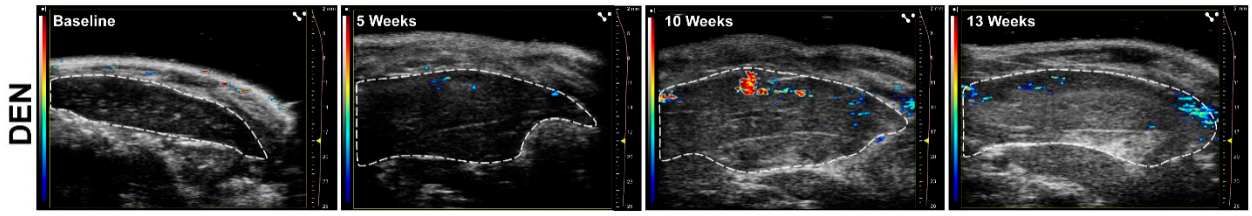

Supplementary Figure S3. PA images of the livers of the DEN-ingested rats. The PA signals obtained are superimposed on the sagittal ultrasound images of the left liver lobe, the borders of which have been outlined. At baseline, the image was made prior to the ingestion of DEN; images were obtained 5, 10, and 13 weeks after DEN administration. The deep red color in the color bar represents the highest (100%) and deep blue color the lowest (0%) PA signal. Subcutaneous PA signals are not observed.

Supplementary Figure S4

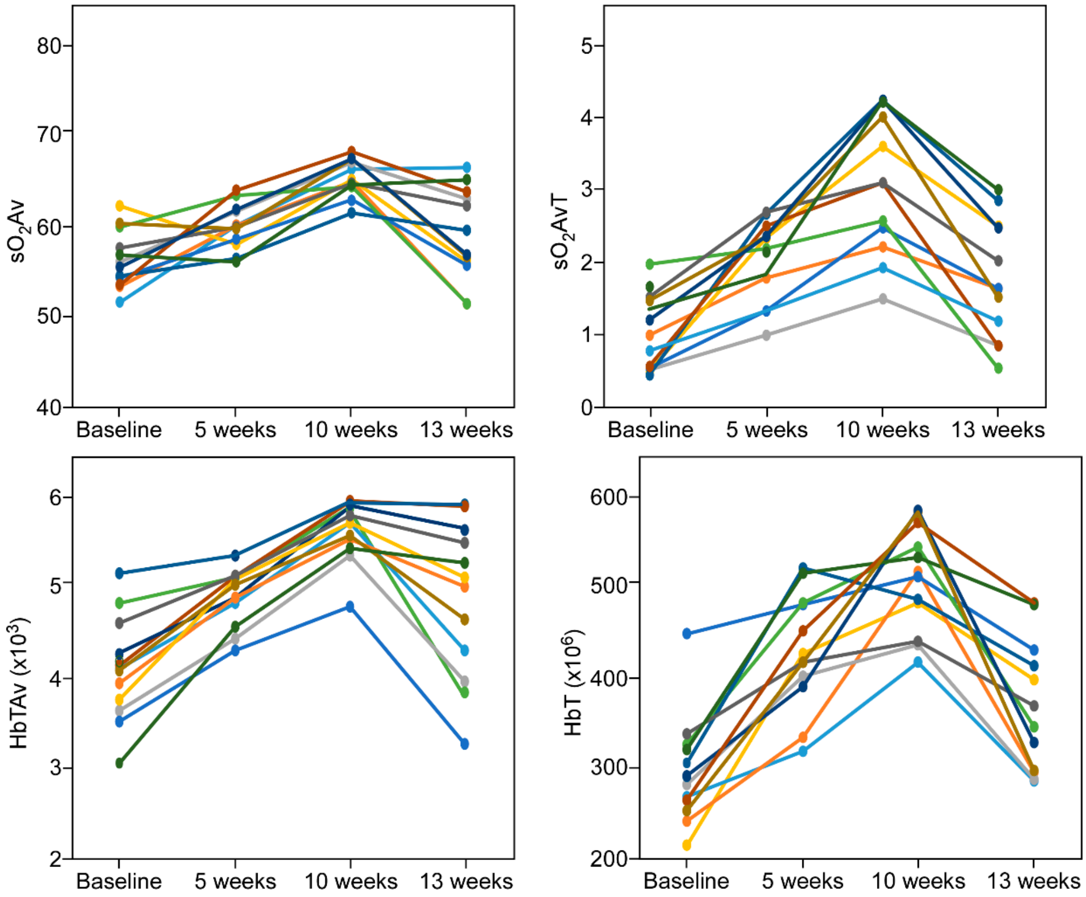

Supplementary Figure S4. Graphs showing hepatic PA signals of individual rats. Values of oxygen saturation (average = sO_2Av and average total = sO_2AvT) and hemoglobin concentration (average = HbTAv and average total = HbT) were obtained for 12 rats (indicated by individual colors) prior to the start of DEN consumption (baseline) and 5, 10, and 13 weeks after DEN administration.

Supplementary Figure S5

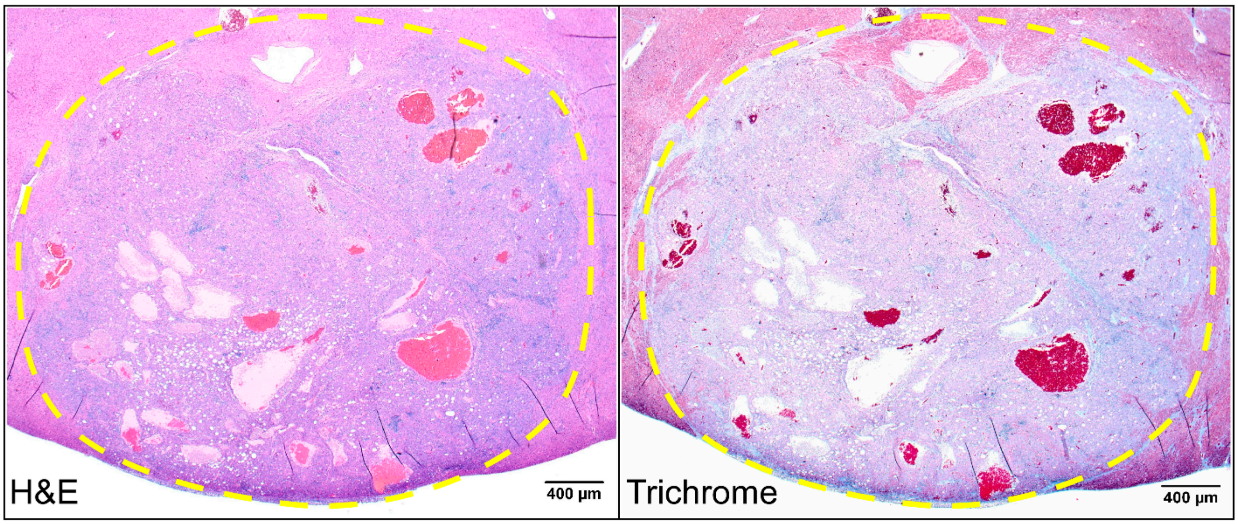

Supplementary Figure S5. H&E (left) and Trichrome (right) staining images showing an HCC tumor in rat liver taken after 13 weeks of DEN ingestion. Area enclosed within the dotted yellow line represents the tumor. Scale bar = 400 µm.
